# How do Positive Deviants Overcome Health-Related Stigma? An Exploration of Development of Positive Deviance Among People With Stigmatized Health Conditions in Indonesia

**DOI:** 10.1177/10497323211058164

**Published:** 2021-12-14

**Authors:** Sarju Sing Rai, Elena V. Syurina, Ruth M. H. Peters, Annisa Ika Putri, Irwanto Irwanto, Marjolein B. M. Zweekhorst

**Affiliations:** 1 Athena Institute, Faculty of Science, 1190 Vrije Universiteit Amsterdam, Amsterdam, The Netherlands; 2 Barcelona Institute for Global Health (ISGlobal), University of Barcelona, Barcelona, Spain; 3 Faculty of Psychology, 64732 Atma Jaya Catholic University, Jakarta, Indonesia

**Keywords:** stigma, chronic illness and disease, power empowerment, adaptation, coping, enduring, southeast Asia, Asian people, grounded theory research strategies, Indonesia

## Abstract

A constructivist grounded theory approach was used to understand how some people living with stigmatized health conditions develop positive deviance to overcome stigma. We examined interviews from 13 identified positive deviants living with four different stigmatized health conditions (HIV, leprosy, schizophrenia, and diabetes) in Indonesia. Positive deviance develops in the form of psychological empowerment through improvement of self-belief and perception (intrapersonal component), development of understanding and skill to exert control in life (interactional component), and self-discovery of successful behaviors and strategies to avert stigma (behavioral component). Positive deviants, after being empowered, start empowering others affected by sharing their knowledge and fostering social awareness and acceptance. The findings revealed the presence of problem-solving ability and agency within the community of stigmatized individuals in Indonesia and warrant researchers to partner with the community to expedite the diffusion of transferable positive deviant strategies within and outside the communities.

## Introduction

Positive deviance is an approach that seeks to solve complex societal problems by uncovering solutions that already exist in the society (Bradley et al., 2009; Marsh et al., 2004). The approach entails identifying individuals called “positive deviants” within the society who have managed to overcome a commonly shared problem by devising their own unique techniques and mechanisms using readily available resources within the community and then learning and replicating those successful strategies to help others in the community (Marsh et al., 2004; Pascale & Monique, 2010). As these unique traits and strategies are derived within the community using resources that are available to all, this approach ensures easy diffusion, uptake, and sustenance of solutions in the community (Marsh et al., 2004; Positive Deviance Initiative, 2010). Studies have highlighted this very scope of positive deviance as an approach to discovering solutions to avert existing health-related adversities in the community (Bradley et al., 2009; Marsh et al., 2004). To date, positive deviance has proven as an effective approach to tackling some of society’s complex health-related problems from child malnutrition (Marsh et al., 2004), smoking cessation (Awofeso et al., 2008), weight loss/control (Stuckey et al., 2011), retention in medical treatment (Assefa et al., 2014), to post-traumatic stress disorder from sexual abuse (Herlof Andersen, 2008).

The positive deviance approach may hold promise in addressing the widely prevalent and complex issue of health-related stigma in the society. Weiss et al. (2006) define health-related stigma as “a social process that instigates devaluation, exclusion and rejection of people based on their health condition.” The experience of health-related stigma among people living with health conditions is broadly categorized into two—enacted and felt stigma. Enacted stigma entails the experience of negative social judgment and discrimination, while felt stigma entails internalized shame associated with having a health condition and fear of experiencing discriminatory acts (Lekas et al., 2011; Scambler, 2004). Health-related stigma is a persistent problem in many communities around the world (Kemp et al., 2019; Sercu & Bracke, 2017; Stangl et al., 2019; Woodgate et al., 2020) and is known to negatively affect the quality of life of people living with stigmatized health conditions (Holzemer et al., 2009; Mosanya et al., 2013; Tsutsumi et al., 2007) and hamper the treatment and management of their disease (Sercu & Bracke, 2017; Stangl et al., 2019). Studies have shown that health-related stigma, particularly felt stigma, can be improved through various stigma reduction strategies and interventions targeted towards people living with stigmatized conditions (Dadun et al., 2019; Lusli et al., 2016; Peters et al., 2016; Rao et al., 2019). For example, Dadun et al. (2019) and Lusli et al. (2016) reported significant reduction in felt stigma among people with leprosy in Indonesia through socio-economic and rights-based counseling interventions respectively. However, existing interventions as such are often cost-intensive and non-sustainable in the long run (Kemp et al., 2019). Many of such stigma reduction interventions involve solutions that reside outside the community, which include resources and practices that require heavy investments and adopter buy-ins, and are often managed by external agents with minimal to non-existent involvement of the community (Kemp et al., 2019; Singhal et al., 2010). Studies have shown that community-based interventions that focus on locally derived and -led solutions can be effective (Bogart & Uyeda, 2009), have greater uptake (Seyfang & Smith, 2007), and likely to be sustainable in the long run (McLeroy et al., 2003).

The concept of positive deviance has prospects in uncovering sustainable and effective solutions to stigma reduction that are self-derived and already exist within the community, ensuring maximal community uptake and ownership. People living with stigmatized health conditions have their own personal agency, and some succeed to overcome stigma in their lives through such self-derived internal mechanisms and strategies (Peters et al., 2014; Shih, 2004). Insights from the life experiences of these positive deviants can help disentangle the unique mechanisms and strategies used by them to mitigate their negative experiences of stigma and help deconstruct their successful approaches to identify those that can be transferred, practiced, and embedded within the community to help others. Understanding positive deviance can not only help uncover and improve understanding on the mechanism of self-agency, but also help in identification of effective, innovative, and sustainable solutions for stigma reduction that may already exist in the community. However, this has, to our knowledge, not been done yet, and still remains an underdeveloped and underexplored area of research.

Therefore, we aimed to explore and understand the process of development of positive deviance to overcome stigma among people living with four distinct health conditions—HIV, leprosy, schizophrenia, and diabetes—in Indonesia. The context of Indonesia as a developing upper-middle income country with high burden of both communicable and non-communicable diseases provides an ideal prospect for this study (IHME, 2010; Kemp et al., 2019; Stangl et al., 2019). Studies have found that Indonesia not only has high burden of infectious diseases, particularly HIV (UNAIDS, 2017) and leprosy (WHO, 2017), and non-communicable mental illness like schizophrenia (HRW, 2016) or a chronic metabolic disorder like diabetes (IDF, 2017), but also rampant stigma and discrimination associated with these four health conditions (Lusli et al., 2016; Pujilestari et al., 2014; Rai et al., 2020; Subu, 2015; Sianturi et al., 2019). Further, studies have indicated that while the cause/origin and severity of stigma may differ across different health conditions, the experience of stigma and their consequences remain largely similar (Rai et al., 2020; Rao et al., 2019; Van Brakel et al., 2019). Taking into consideration such similarities that exist in stigma experiences across different health conditions, we sought to explore the pathway in which people living with one of these four stigmatized diseases in Indonesia develop positive deviance to overcome stigma.

### Positive deviance approach for social change

“Positive deviance (PD) is an approach to social change that enables communities to discover the wisdom they already have, and then to act on it” (Pascale & Monique, 2010).

The positive deviance approach strives for sustainable behavioral and social change through identification of solutions that are community-derived, diffused and owned (Positive Deviance Initiative, 2010; Van Dick & Scheffel, 2015). There are many methodologies for the positive deviance approach (Marsh et al., 2004; Positive Deviance Initiative, 2010; Pascale & Monique, 2010). Nonetheless, the characteristics of the different methodologies are very similar. All methodologies acknowledge the importance of identifying the needs of the community, the problem, and the positive deviants. Furthermore, all methods try to discover successful behaviors and/or practices of the positive deviants; this is often called the positive deviance inquiry (Positive Deviance Initiative, 2010). The only difference exists in the final stage of the process, where the steps can lead to either monitoring and evaluation (Marsh et al., 2004; Positive Deviance Initiative, 2010) or continuous research on the positive deviants within a community and extend the positive deviance research to other communities (Fowles, 2007).

One of the widely used methodologies is the one proposed by the Positive Deviance Initiative (Positive Deviance Initiative, 2010). This methodology consists of five basic steps, namely, (i) defining the problem; (ii) determining the presence of positive deviants (usually four to six people, as they are rare); (ii) discovering successful behavior that offers solution to the problem; (iv) designing activities using the discovered solution; and (v) monitoring and evaluation. The first four steps are also known as the four Ds (Positive Deviance Initiative, 2010). Within the scope of this study, only the four Ds of the positive deviance methodology were utilized, with focus on assimilating the findings and recommending subsequent actions for the fourth D instead of designing activities.

## Methods

### Study Settings and Procedure

This study was a part of an exploratory community-based study on health-related stigma conducted in Jakarta and west Java in Indonesia between March and June 2018 among people with/affected by either of the four stigmatized health conditions—HIV, leprosy, schizophrenia, and diabetes. This study comprised of an exploratory qualitative design to understand the process of development of positive deviance. These four specific health conditions were chosen because of their higher prevalence (MOH, 2013; IDF, 2017; UNAIDS, 2017; WHO, 2017) in comparison to other south-east Asian nations and stigmatization in the Indonesian society (Lusli et al., 2016; Peters et al., 2013; Pujilestari et al., 2014; Subu, 2015; Sianturi et al., 2019). Participants were recruited purposively from the community through referrals from community-based organizations and peer-support groups. Those who were over the age of 16 (age of consent) and who were willing and consented to participate were included in the study. This study was based on in-depth interviews.

### Selection of Positive Deviants

A multi-stakeholder consultation with local researchers, community leaders, peer leaders, and representatives of community-based organizations was undertaken to identify positive deviants across the four conditions. The role of the stakeholders in this study was limited to the identification, selection, and recruitment of the participants. As per the consultation, a four criteria definition for positive deviants was derived. Positive deviants were those who (i) successfully managed to overcome stigma in their lives, (ii) were not ashamed of their disease, (iii) innovated unique mechanisms and ways to avert stigma, and (iv) managed to reintegrate into the society. A two-step identification and recruitment of positive deviants was then conducted. In the first step, 21 individuals (HIV: 6; leprosy: 6; schizophrenia: 5; diabetes: 4) were identified through referrals from local resource persons. This took place in different ways for each condition: possible positive deviants with leprosy were identified in Cirebon through recommendations from peers and resource persons from local leprosy-related organization, those with HIV and schizophrenia were identified through peers and community-based organizations in Jakarta, and those with diabetes were recruited through peer-recommendation and patient support groups in Jakarta. In the second step, the identified positive deviants were further confirmed through preliminary in-depth interviews using the aforementioned criteria. Based on the interviews, 13 positive deviants were finally selected (HIV: 4; leprosy: 3; schizophrenia: 4; diabetes: 2). A final consultation was conducted among the research team and community stakeholders for consensus on the selection of positive deviants.

### Data Collection

Qualitative semi-structured in-depth interviews were conducted with identified positive deviants. Informed consent was sought prior to participation in the study. Those who agreed were either interviewed in their homes or at the NGO offices in privacy. Prior to interview, sociodemographic information on age, gender, ethnicity, religion, income, etc. was collected from the participants. The interviews started in an exploratory manner and progressed toward more in-depth questions to uncover the participants’ self-reported trajectory/process of positive deviance. On average, the interviews lasted an hour. During the interviews, a guide was used (Supplementary File 1). The following topics were addressed by the interview guide to uncover the development of positive deviance: general information, life history, experience of living with the health condition, experience of stigma and discrimination, turning point in life, strategies used to avert stigma, perceived changes after overcoming stigma, and current works and activities. Focused probing was conducted to discover successful strategies and behavior employed by participants to mitigate stigma in their lives. The interviews included exhaustive probing along with member check/respondent validation to ensure trustworthiness of the collected data. The interviews were recorded, transcribed, and translated into English. Data management and analysis were performed using Atlast.ti software.

### Data Analysis

Data analysis was independently carried out by the two co-authors—one from the field of global health (Sarju Sing Rai) and other from psychology (Elena Syurina). The obtained codes were discussed among the authors: common codes were combined, while the different and discrepant codes were further deliberated on and noted. All such codes/categories were discussed with and agreed upon by all co-authors in each stage of the analysis to make final decisions on the codes/categories. The constructivist grounded theory approach outlined by Charmaz (Charmaz, 2014) was used for data analysis. Using Charmaz’s approach, data analysis entailed an extensive process of coding and memo writing. The analysis was conducted as follows:

### Initial Coding

The transcripts were read line-by-line and incident-by-incident coding was conducted within each interview to obtain the initial codes.

### Focused Coding

After obtaining the initial codes, they were compared across the interviews from all positive deviants. Codes were first clustered into categories based on the two preliminary evolving themes that emerged from analysis—development of positive deviance and impact of positive deviance. As the coding process proceeded, it became clear that some of the categories corresponded to most positive deviants, which became the main theoretical categories. There were some categories that were less mentioned and formed sub-categories. The codes were then categorized and preliminary categories were obtained.

### Theoretical Coding

In the theoretical coding process, the categories obtained through focused coding from all 13 interviews were further studied, compared, and organized. The process entailed interlinking and synthesizing of those categories that led to integration of interrelated categories into themes. Three main themes emerged from theoretical coding, viz. “triggers” that led to positive deviance, “empowerment” as an actual process of positive deviance, and “impact” of positive deviance. Under each theme were interrelated categories and sub-categories. The process of theoretical coding led to a preliminary theoretical framework obtained through assimilation and thematic conceptualization of interrelated categories.

### Application of Extant Theoretical Models

As per the constructivist grounded theory approach, the resultant theoretical concepts were compared with existing relevant literature in the later stage of data analysis. The theoretical categories pertaining to the theme “empowerment” was found to bear strong similarity to the existing theoretical models of psychological empowerment. Upon further review and comparison, the authors deemed fit to integrate the theoretical concept of “empowerment” into the existing theoretical model of psychological empowerment by Zimmerman (Zimmerman, 1995).

Zimmerman’s psychological empowerment model (Zimmerman, 1995) was used to describe the process of development of positive deviance. The model posits that psychological empowerment entails three components, viz. intrapersonal, interactional, and behavioral component, which all work together in unison to empower an individual. The first component—intrapersonal empowerment—refers to how individuals think about themselves and perceive control, self-efficacy, and motivation in their lives. This component is about the individual’s perception of self-esteem, self-view, and locus of control. The second component—interactional empowerment—refers to the critical awareness and understanding people have about their condition, community, and society (norms, structures, etc.), and the ability and skillset individuals have to make a change. This component posits that individuals need to learn about their options and scenario in a given context in order to be able to exert control in their environment. The final component—behavioral empowerment—occurs when individuals have both intrapersonal and interactional empowerment to drive a positive behavior change. Behavioral empowerment refers to actual actions/behaviors/strategies undertaken to make a change and directly influence outcomes in one’s life (Zimmerman, 1995).

### Memo Writing

Memo writing was a crucial part of the entire data analysis process as outlined by Charmaz (Charmaz, 2014). The authors wrote down memos on the evolving codes and resultant categories and themes in every step of the analysis. The memos helped in thorough reflection, comparison, and elucidation of the emerging codes and categories and guided the whole analytical process.

### Final Theoretical Framework

A final theoretical framework of the process of development of positive deviance was obtained after integration of the main themes with the extant theoretical model. The final theoretical model includes four thematic stages that underline the process of development of positive deviance among the participants.

### Rigor of Study

The analytical process was peer debriefed and discussed in each stage to ensure the quality and trustworthiness of codes, categories, and themes. The authors made sure all codes, categories, and themes were obtained until the point of saturation. The results of the analysis were carefully reviewed and refined in all stages. Rigorous vetting and deliberation were carried out among the authors in determining the causal and consequential relationships between the categories and developing the preliminary theoretical framework from thematic coding, and then integrating the theoretical concepts into existing theoretical models after literature review. The final framework was thus obtained after consensus from all authors on the final themes and the logical connection and pathway they describe. An outline of the analytical process and resultant outcomes in each stage of analysis is presented in Supplementary File 2.

### Ethical Approval

This study was performed in line with the principles of the Declaration of Helsinki. Approval was granted by the Ethics Committee of Atma Jaya Catholic University, Indonesia (Approval ID: FR-UAJ-26-13/R0). All participants provided verbal and written consent to participate in the study.

## Results

### Demographic Information About Participants

A total of 13 individuals were identified as positive deviants. Out of them, four were living with HIV, three were affected by leprosy (two with visible physical impairments), four with schizophrenia, and two with diabetes (with no diabetic complications and resulting impairments). There were six male and female respondents, while one identified as transgender. The majority of the respondents belonged to Jawa and Sunda ethnicity (67.6%), and followed Islam (69.2%). The median age of the positive deviants was 41.31 years (SD 14.22) with an age range between 20 and 75. The mean duration of living with/having had the condition was 12.31 years (SD 5.99) with a range between 1 and 25 years.

### The Process of Development of Positive Deviance

**Figure 1.**
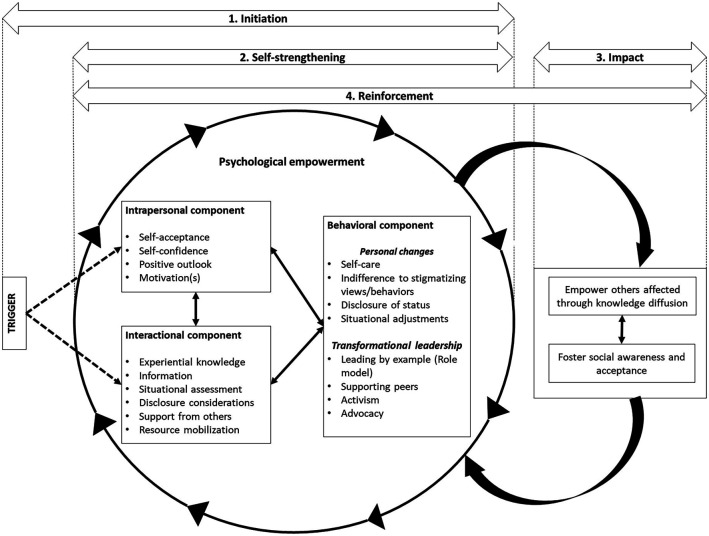
The process of development of positive deviance.

The final theoretical framework obtained from the constructivist grounded theory analysis described four stages of the process of development of positive deviance among the participants: (i) initiation, where specific triggers helped initiate the process of psychological empowerment among positive deviants, (ii) self-strengthening, where the process of psychological empowerment repeated in a loop while further strengthening its components, (iii) impact, where after being empowered, positive deviants started helping others and contributing to the society, and (iv) reinforcement, where the process of impact and psychological empowerment repeated in a reiterative cycle, enriching each other in every iteration. The framework illustrating the four stages of development of positive deviance is outlined in Figure 1.

## Initiation

Theoretical coding showed that participants developed positive deviance through psychological empowerment. The process of psychological empowerment adapted from Zimmerman’s model (1995) constituted three components:

a. Intrapersonal component: Positive deviants reported of intrapersonal component of psychological empowerment that helped them improve their self-belief and perception and led them to take charge of their lives. The categories of intrapersonal component included: (1) “self-acceptance,” wherein positive deviants talked about how accepting themselves and their health condition was the first step to feeling better about themselves and improving self-esteem; (2) “self-confidence,” which played an important role for them to be self-aware of their ability to perceive control of their life; (3) “positive outlook,” wherein positive deviants reported of having positive insights and outlook to life, which ignited a sense of hopefulness and normalcy in their lives; and (4) “motivation,” which provided them the incentive to keep going on and striving for better.

Motivation was further categorized into three sub-categories based on its sources: (i) “empathy towards other stigmatized individuals,” where motivation came from being able to relate to others living with the same health condition as them and having empathy towards them; (ii) “experience of stigma/discrimination,” wherein motivation came from the experience of stigma in their everyday life from family, friends, and healthcare professionals; and (iii) “concern for loved ones,” where motivation came from the concern that positive deviants had for their loved ones—especially close family members (children, spouse, father, mother, etc.).

b. Interactional component: Positive deviants reported of interactional components that helped them develop critical awareness and understanding of their disease and the environment, and gain skills to manage stigma. This included (1) “experiential knowledge,” wherein positive deviants reported of how their personal experiences of living with the health condition provided a better understanding of not only their disease but also the context of stigma surrounding them; (2) “information,” wherein they talked about how gaining relevant knowledge and information, either from other experts or through information resources, helped them understand their disease and think of ways to manage them; (3) “situational assessment and awareness,” in which they talked about the importance of having a critical awareness of the environment and the social norms in one’s daily life, and understanding measures to fit-in as a way to avert stigmatization; (4) “disclosure considerations,” wherein they highlighted the importance of understanding the people, their beliefs and specific contexts before disclosing one’s health status to others; (5) “support from others,” where they talked about how perception of support from others, especially family and friends, provide positive reinforcement in their lives; and (6) “resource mobilization skills,” wherein positive deviants discussed how critical awareness of available resources or ways to accessing different resources is paramount to effective management of their life.
c. Behavioral component: Behavioral component in this study entails successful behavior changes exhibited by the positive deviants to overcome stigma. Positive deviants reported of two different concepts of behavior change—“personal changes” that helped them in their everyday life to overcome stigma, and ‘transformational leadership’ in which they started helping others affected to fight against stigma.

Personal changes included behaviors like (1) “self-care,” wherein positive deviants highlighted the importance of caring for themselves for not only effective management and treatment of their disease but also for overall well-being. Self-care entailed both physical/medical care (taking medications regularly, following through with health check-ups, etc.) and spiritual care (meditation, prayers, etc.); (2) “indifference to others’ stigmatizing views/behavior,” wherein they talked about how they ignored and did not care about what others thought of them or talked about them. By this point, they perceived enough control of their life and environment that the public views did not affect them; (3) “disclosure of one’s status,” wherein disclosure of their health status was a very important action for positive deviants, which they said not only helped them feel liberated from shame and stigma associated with their disease, but also helped garner support and understanding from others; (4) and “situational adjustments to assert control of the environment,” where they talked about how adjusting according to different contexts, cultures, and situations help them avert stigma and judgment in their daily lives. They highlighted the importance of critical awareness of the environment and norms in helping decide the required adjustments needed.

Transformational leadership included behaviors like (1) “leading by example (being a role model),” wherein having understanding and empathy towards others living with their health conditions, positive deviants discussed how they started helping others by showcasing their own life experiences of living with the condition and mentoring and guiding them; (2) “helping and supporting peers,” where positive deviants actively helped others affected through actions like home visits to track their treatment, emotional support and counseling, connecting/referring them to relevant people within their network, lending money when in need, etc.; (3) “activism,” wherein they talked about their passion to fight for their rights and those of other people affected. They showed aversion to inequality and injustice and actively spoke against those, while demanding equality and justice; and (4) “advocacy,” wherein some positive deviants reported of going a step further ahead from activism towards advocacy where they systematically worked with different stakeholders on affecting policy changes to improve the condition of people living with the health condition as them.

A summary of the categories of the three aforementioned components, their description, and illustrative quotes are provided in Supplementary File 3.

### Role of Triggers in Initiating Psychological Empowerment

Positive deviants were found to already have different levels of intrapersonal and interactional component, as they had already gone through various experiences and exposures in their lives to provide for these two components of empowerment. However, to initiate the process of psychological empowerment, the two components needed to lead to behavioral component (i.e. to initiate behavioral changes to avert stigma). For this to happen, a “trigger” was necessary, which the participants called “the turning point in their life.” Triggers were found to act like sparks that started the process of psychological empowerment by either driving interpersonal or interactional component that ultimately led to behavior change.

For positive deviants who had adequate interactional component but lacked sufficient intrapersonal component, a trigger in the form of added motivation helped in initiating the empowerment process. The motivational trigger came in different forms. For some positive deviants, motivation came after remission of their disease or improvement of their health after treatment. For others, motivation took shape in the form of indignation and revolt against discrimination. One positive deviant living with HIV gave an account of how at first she was complacent and tolerated discrimination from others, but when it was directed to her loved ones—she revolted: *“[…] as long as the discrimination was only directed to me... […] I just accepted it. But…when I saw people close to me getting discriminated against, I got angry. […] my husband needed to get operated and we got denied from hospital […]. I ended up getting really angry. I reported it to ministry of health and I took it to the media. I ended up meeting the president of the hospital because I also didn’t want it to get to the media. (Female, HIV)”*

In case of positive deviants with adequate intrapersonal component but not enough interactional component, a trigger in the form of information that built their knowledge and skill helped strengthen interactional empowerment to drive behavior change and initiate the process of positive deviance. Information received through internet, social media, friends, family, healthcare professionals, and other individuals living with the condition (peers) helped ignite their passion to make positive change(s) in their lives. One positive deviant living with HIV who was in prison when tested positive for HIV recounted his experience of how information helped trigger positive deviance in his life: *“The information that I got inside (the prison) … about HIV was limited. […] So, I called my friends and told them about my status, wishing they could look for books [on HIV]. But it turns out there were none, it was not that easy. […] I could only look at it from google, but Information that I got were limited. I tried to look for more information…luckily, there was a doctor from America who was conducting research inside the prison, so he/she told us […] as long as you have HIV, you could get medication to treat HIV. Knowing that, I fought for my rights and started arguing why we needed to wait until our condition got worse, if we were ready! (Male, HIV)”*

Triggers initiated psychological empowerment in the lives of the respondents in different stages of their lives. Some positive deviants encountered triggers at the earliest point when they came to know of their disease status, while for some it took a longer time—until a specific event or experience drove them to make changes in their lives. Further, the strength of triggers also differed as per individuals. For some, triggers had to be strong and cathartic to catalyze positive change like a traumatic experience of discrimination, while for some, they were very mild and gentle, much like an epiphany.

## Self-Strengthening

Once initiated, the process of psychological empowerment was found to strengthen further by functioning in a reiterative loop, with each empowerment component leading to strengthening of either of the other two components, and so forth (see Figure1). One positive deviant with schizophrenia described this process which started with him recovering and feeling better and confident (intrapersonal empowerment), which led him to actively search for information and resources regarding his condition and learning ways to improve it (interactional empowerment), finally leading to making important changes in his life to improve his condition (behavioral empowerment): *“I recovered (from schizophrenia) and I started looking more into what was I sick with. I looked it up (in google) and I learned that the symptoms matched with mine. But I did not stop there and started looking for more. I created my Facebook account […] and I found KPSI (NGO on Schizophrenia) and contacted them. After meeting others like me I was then sure that the pills really do help with our recovery. So, I regularly took my pill from 2010 until now. Thank God I haven’t relapsed since then. (Male, Schizophrenia).”* Positive deviants, once entered into the process of psychological empowerment, were found to undergo the process reiteratively further building up on each of the three components that led to self-strengthening with time.

## Impact

With initiation, development and strengthening of psychological empowerment, the positive deviants ultimately sought to bring about two distinctive impacts—directed toward others living with their health condition and toward the outside world. The first type was to strengthen and empower other stigmatized individuals from their group through “knowledge diffusion.” Positive deviants reported of how they were able to help others understand their disease and ways to manage it and avert stigma and other adversities in life. They talked about sharing with others the behaviors and strategies they used in their own lives, which helped them. One positive deviant with HIV talked about the advice he gives to others living with HIV: “*I feel happy that I have been able to help others with HIV. I always tell them that accepting yourself first is the most important thing. If you can't accept yourself, how will others accept you. If you are able to accept yourself, others will follow. Even if others can’t accept you, at least you can accept yourself. (Male, HIV).”*

The second impact was to “foster wider social awareness and acceptance” of people living with the condition. Positive deviants talked about their yearning for acceptance and inclusion in the society, for which they made sure to portray a positive picture of how people with health conditions as them are just like any other person and are a productive member of the society. One positive deviant with HIV explained: “*[…] I always try to show people that despite of having HIV, I am a productive person who contributes to the society. People will set aside the fact that you have HIV…. even if you’re gay and LGBT ….if they see you have skills and are productive. I taught in some government agencies and they don’t care that I have HIV, they just appreciate what can I give to the company. So that’s what I always emphasize “what can you give to other people?”. If you do good things, people will respect and appreciate you. (Male, HIV).”*

## Reinforcement

Successfully achieving impact was found to further encourage and reinforce the positive deviants’ psychological empowerment, which in turn encouraged them to strive for more impact—thus creating a reiterative positive loop between the process of psychological empowerment and the resultant impact (as illustrated in Figure 1). Positive deviants talked about how they felt more driven and motivated and strived to work harder to help others after seeing the positive impact they have been able to bring in the community and to people’s lives. One positive deviant with leprosy said in regards to helping others affected by leprosy: *“When I saw how I was able to motivate other people with leprosy, I became even more motivated. I started learning new skills and working with other villagers to improve our village infrastructure. I showed everybody that even though I have leprosy, I can positively contribute to the society. Seeing my work, my friends with leprosy are even more motivated. … (Male, Leprosy).”* Another positive deviant with HIV talked about how he managed to show the public that *“people with HIV can equally contribute in the society”* by teaching aerobics to the police, and reported receiving a lot of support from the public, which further fueled his desire to do more to improve their understanding and perception towards people living with HIV in the society.

## Discussion

Through this study, we attempted to explore and understand the process of development of positive deviance among people living with stigmatized health conditions in Indonesia as a way to avert stigma. Positive deviants exhibited psychological empowerment triggered by their unique life events and experiences. Once initiated, the process of psychological empowerment was found to self-strengthen by functioning in a reiterative loop—with each empowerment component leading to strengthening of either of the other two components and so forth. With initiation and self-strengthening of psychological empowerment, the ultimate impact that the positive deviants were trying to bring about were empowerment of other stigmatized individuals from their group through diffusion of successful strategies in the community and striving for social change by fostering wider social awareness and acceptance of people living with the condition. The processes of impact and psychological empowerment were found to repeat in a reiterative cycle, enriching and building upon each other in every iteration.

Positive deviance developed in the form of psychological empowerment which helped individuals overcome the experiences of stigma and related adversities in everyday life. Depending on the field of research and the setting, positive deviance can take different forms (Herlof Andersen, 2008; Marsh et al., 2004). Among Vietnamese farmers facing the issue of child malnutrition, positive deviance came in the form of going against the community tradition and diversifying the children’s diet to include other macronutrients other than just carbohydrates (Marsh et al., 2004). For men dealing with childhood experience of sexual abuse, positive deviance took shape in the form of focusing and building on a positive identity that identifies less with the experience of abuse (Herlof Andersen, 2008). In this study, psychological empowerment was indicative of positive deviants’ ability to perceive control in their life (interpersonal empowerment) and develop understanding of the social contexts and systems and ways to maneuver them (interactional empowerment), which enabled them to exert required actions to bring about positive changes in their lives that helped them overcome stigma and related adversities (behavioral empowerment) (Zimmerman, 1995).

Further, once the process was initiated, the three components of psychological empowerment reiteratively self-strengthened each other while also leading the positive deviants to make an impact by empowering others and fostering public awareness. This in turn further strengthened the process of psychological empowerment among positive deviants that again led to more impact—thus creating a positive reiterative loop. This is consistent with Fredrickson’s broaden-and-build theory (Fredrickson, 2001) which posits that positive psychological processes often enhance and grow in each thought-action repertoire creating an upward spiral. Studies on positive psychology have demonstrated this particular phenomenon where positive processes like coping with adversities, psychological resilience, and emotional well-being keep building and growing in a cyclical manner with each positive thought-action repertoire (Aspinwall, 1998; Fredrickson & Joiner, 2002).

The findings indicate that psychological empowerment can be an effective way to overcome stigma among people living with stigmatized health conditions. This corroborates with the works of Oyserman and Swim (2002) and Shih (2004) which posited that empowerment can help stigmatized individuals overcome adversities caused by stigma, build self-efficacy, and reject negative labels associated with their stigmatizing trait. This may be a possible mechanism that can lead to reduction of felt stigma. Hence, interventions focused on psychological empowerment may have prospects in stigma reduction among those living with different health conditions. However, there may be two different approaches to designing such interventions. The first approach is the conventional etic empowerment approach led by external agents (researchers, program managers, etc.) whereby specific interventions are designed and introduced targeting the different components of psychological empowerment (Chopra & Ford, 2005; Sadan, 1997). Studies have indicated that such interventions can be effective (Eisman et al., 2016; Peterson & Reid, 2003). Eisman et al. found that targeting the three empowerment components not only improved the overall psychological empowerment, but also increased the likelihood of positive behaviors among youth (Eisman et al., 2016). Further there have been stigma reduction interventions focused on empowering people living with stigmatized health conditions, which have shown promising results in reducing felt-stigma (Dadun et al., 2019; Ebenso et al., 2007; Peters et al., 2016). However, studies have found that such interventions are often resource-intensive, have poor uptake by target beneficiaries, and not sustainable on the long run (Singhal et al., 2010; Singhal & Svenkerud, 2019; Stangl et al., 2019). This may not only have happened because of the obvious reason that the etic approach is often designed and led by external agents, but also because such interventions extensively focus on successful strategies which may work only in context of certain individuals and settings, and not the wider community.

It is important to understand that for individuals like positive deviants, psychological empowerment is a self-derived mechanism, while for others, it might not be as obviously feasible. It can therefore be beneficial to follow a second approach—the positive deviance approach (Marsh et al., 2004; Positive Deviance Initiative, 2010; Pascale & Monique, 2010) that focuses in identifying the most usable and transferable behaviors or strategies employed by positive deviants that can be easily implemented, practiced, and shared by others in the community, which may help lead those who are not positive deviants into the path of psychological empowerment. For example, strategies such as selective disclosure of one’s health condition can be emulated by people living with stigmatized health conditions. As opposed to full disclosure, selective disclosure entails disclosing of one’s health condition to a selected group of people who the stigmatized individual trusts and/or expects understanding and support from (Corrigan & Rao, 2012). While this strategy may not help individuals fully mitigate stigma as feelings of shame and apprehension may still exist, it is known to provide a needed reprieve to stigmatized individuals by gaining supporters/confidants and getting a sense of control of how, when and who they disclose their health condition to (Black & Miles, 2002; Corrigan & Rao, 2012). This strategy has already proven effective among people living with HIV (Black & Miles, 2002; Kumar et al., 2015) and mental illness (Bos et al., 2009; Corrigan & Rao, 2012) in coping with stigma. Hence, such positive deviant strategies have prospects for broader acceptance, ownership, and practice in the community as they are derived from people from the community with similar experiences and contexts (Pascale & Monique, 2010). The positive deviance approach may thus have the answer to designing future stigma interventions that are not only effective but also sustainable.

Positive deviants living with stigmatized conditions had passion and drive to help others in the community and yearned to make an impact among both other stigmatized peers and the society. Besides helping other stigmatized individuals, becoming role models, and advocating for their rights, they shared personal stories and specific strategies in their community. This may have already led to a diffusion of innovation (transference and transformation of tacit knowledge to implicit knowledge) within their community. Studies on psychological empowerment have shown that people who are empowered tend to empower others (Eisman et al., 2016; Sadan, 1997; Zimmerman, 1995). Eisman et al. (Eisman et al., 2016) found that psychological empowerment among youths in turn helped increase their prosocial involvement and contribution to the community. Positive deviants were further involved in advocacy to help other oppressed stigmatized individuals. This is consistent with the findings from Sadan (Sadan, 1997) who noticed that empowered individuals focused on advocacy to ensure even the weakest people can access empowerment process by creating the minimal environmental conditions.

Further, they strived for social change and were known to put themselves out in the public eye to foster understanding and awareness of their condition. They aimed to elicit public understanding and empathy toward themselves and other people living with their condition—paving the way to social acceptance, reintegration, and possible inclusion. Sadan (Sadan, 1997) noted similar behavior among empowered people where empowerment extended beyond personal context like individual achievements—toward a path of social transformation. On discovering one’s right and ability to control one’s destiny, empowered individuals were known to systematically change their lives and environment, and consequently strive for social and political change (Wallerstein & Bernstein, 1994; Sadan, 1997).

The findings from this study show prospects of the positive deviance approach to catalyze social change from within the community and provide comprehensive information on the “know-how” for those affected to empower themselves and overcome stigma and its negative effects. However, it is also important for the external agents and environment to also facilitate the process of social change. As per the social change theory it is important to influence both the normative and social structures in order to bring about social transformation (De La Sablonnière, 2017). The positive deviance approach is mostly focused in the normative level (Pascale & Monique, 2010; Singhal et al., 2010). While the approach may extend to exert influence on the social structural level (Marsh et al., 2004), the attempt may still fall short. In order to deal with a deeply rooted and complex issue of health-related stigma, it is important for allies from outside of the community to help in efforts to effect change in the social structural level (Singhal et al., 2010; De La Sablonnière, 2017; Singhal & Svenkerud, 2019).

In this context, researchers, community leaders, and health program managers have an important role within the positive deviance approach that first and foremost includes understanding and acknowledging the existence of problem-solving ability and innovation within the community of stigmatized individuals. This is followed by partnering with the community members in identifying positive deviants and their successful strategies that can be used with ease within the community, and wider diffusion of those strategies. Researchers can help expedite this process of diffusion of innovation within the community. Further, researchers can play a very important role in the cross-diffusion of such successful strategies from one community to another, and subsequent participatory monitoring and evaluation of the process of social change. It is important for researchers and program managers to build an equitable alliance with the community members and support positive deviants as role models and leaders in the community. Further they can also play an important part in linking the community with other relevant stakeholders and advocacy partners to establish a united front to address both the normative and social structural level changes needed for stigma reduction in the society.

This study also has certain limitations. While we uncovered how the process of self-strengthening and impact reiteratively reinforce each other, we could not determine the extent of the reiterations, specifically if such processes keep on building up throughout the whole life of the positive deviants or if they plateau at some point in life. Future studies should explore this further. Further, this study does not take into account the differences in the origin and severity of stigma across different health conditions, but rather focuses on the overall process of overcoming stigma. It is therefore important to take these unaccounted factors into consideration while formulating stigma reduction strategies based on the findings from this study. Despite the limitations, this study has several notable strengths. This study was able to identify 13 positive deviants through rigorous vetting. Marsh et al. (Marsh et al., 2004) have recommended a sample size of four to six participants in research involving the positive deviance approach owing to the rarity of positive deviants in the community. However, by partnering with the community stakeholders, we were able to identify and verify a much larger number of positive deviants than expected. To our knowledge, this was the first study to use the positive deviance approach to explore and understand how some individuals manage to overcome health-related stigma. Future studies should further expand on this approach by partnering with the community as an ally to design activities or interventions that can help other stigmatized individuals overcome stigma. Further, it is recommended that the positive deviant behavior and strategies identified in the study be utilized in stigma reduction interventions to assess its transferability, translationality, and effectiveness in non-positive deviants living with stigmatized health conditions.

## Conclusion

The positive deviance approach employed in this study to understand how positive deviants overcome stigma demonstrates the presence of problem-solving ability and agency within the community of stigmatized individuals in Indonesia. Positive deviance took shape in the form of psychological empowerment. The process of how positive deviance develops among people living with stigmatized health conditions and the successful behavior they employ to avert stigma in their lives may hold promise in helping others affected by stigmatized diseases in Indonesia in overcoming stigma and fostering social inclusion through the process of psychological empowerment. Positive deviants have been crucial in not only coming up with these unique solutions and strategies to avert stigma, but also diffusion of those through knowledge sharing and support to other stigmatized individuals. Further, they strive for social change by fostering awareness and relatedness, and changing the perception of the societies’ way of viewing people living with health conditions. Researchers can play an important role in this process by partnering with the community and helping expedite the process of diffusion of positive deviant strategies within and outside the communities.

## Supplemental Material

sj-pdf-1-qhr-10.1177_10497323211058164 – Supplemental Material for How do Positive Deviants Overcome Health-Related Stigma? An Exploration of Development of Positive Deviance Among People With Stigmatized Health Conditions in Indonesia Click here for additional data file. Supplemental Material, sj-pdf-1-qhr-10.1177_10497323211058164 for How do Positive Deviants Overcome Health-Related Stigma? An Exploration of Development of Positive Deviance Among People With Stigmatized Health Conditions in Indonesia by Sarju Sing Rai, Elena V. Syurina, Ruth M. H. Peters, Annisa Ika Putri, Irwanto Irwanto and Marjolein B. M. Zweekhorst in Qualitative Health Research

sj-pdf-2-qhr-10.1177_10497323211058164 – Supplemental Material for How do Positive Deviants Overcome Health-Related Stigma? An Exploration of Development of Positive Deviance Among People With Stigmatized Health Conditions in Indonesia Click here for additional data file. Supplemental Material, sj-pdf-2-qhr-10.1177_10497323211058164 for How do Positive Deviants Overcome Health-Related Stigma? An Exploration of Development of Positive Deviance Among People With Stigmatized Health Conditions in Indonesia by Sarju Sing Rai, Elena V. Syurina, Ruth M. H. Peters, Annisa Ika Putri, Irwanto Irwanto and Marjolein B. M. Zweekhorst in Qualitative Health Research

sj-pdf-3-qhr-10.1177_10497323211058164 – Supplemental Material for How do Positive Deviants Overcome Health-Related Stigma? An Exploration of Development of Positive Deviance Among People With Stigmatized Health Conditions in Indonesia Click here for additional data file. Supplemental Material, sj-pdf-3-qhr-10.1177_10497323211058164 for How do Positive Deviants Overcome Health-Related Stigma? An Exploration of Development of Positive Deviance Among People With Stigmatized Health Conditions in Indonesia by Sarju Sing Rai, Elena V. Syurina, Ruth M. H. Peters, Annisa Ika Putri, Irwanto Irwanto and Marjolein B. M. Zweekhorst in Qualitative Health Research
