# Supplementary material for: How do Positive Deviants Overcome Health-Related Stigma? An Exploration of Development of Positive Deviance Among People With Stigmatized Health Conditions in Indonesia
Source: Qual Health Res. 2021 Dec 14;32(4):622–34. doi: 10.1177/10497323211058164 (PMC8853968; doi:10.1177/10497323211058164)
Supplement: sj-pdf-1-qhr-10.1177_10497323211058164 – Supplemental Material for How do Positive Deviants Overcome Health-Related Stigma? An Exploration of Development of Positive Deviance Among People With Stigmatized Health Conditions in Indonesia [file sj-pdf-1-qhr-10.1177_10497323211058164.pdf]

SUPPLEMENTARY FILE 1

Interview Guide

This semi-structured interview guide was used to steer the in-depth interviews in which the positive deviants were asked to self-report the process of development of positive deviance in their life by detailing their lived experiences. The following topics are addressed by the interview guide to uncover the development of positive deviance: general information, life history, experience of living with the health condition, experience of stigma and discrimination, turning point in life, strategies used to avert stigma, perceived changes after overcoming stigma, current works and activities, and future.

- Did you ever feel stigmatized and discriminated against because of your health condition (HIV, leprosy, diabetes, schizophrenia) ? Please tell me about your experiences of living with your health conditions – any stigma and discrimination you faced because of your health condition.

  Probe:

  - *Think about the time when you first found out about your disease. How has your life changed after you found out about your condition?*
  - *What have your experiences been of living with the condition: (i) Within your family life (nuclear family or extended family), (ii)At work/school, (iii) Within your religious community (if any), (iv) At the health facility (Probe further: Tell me more. Can you give an example? Another example?)*

- Do you still feel stigmatized and discriminated against because of your health condition (HIV, leprosy, diabetes, schizophrenia) ? Please tell me about your recent experiences (within the last 6 months) of living with your health conditions.

  Probe:

  - *What have your experiences been recently of living with the condition: (i) Within your family life (nuclear family or extended family), (ii)At work/school, (iii) Within your religious community (if any), (iv) At the health facility (Probe further: Tell me more. Can you give an example? Another example?)*

- How did your experiences change? What happened in your life that helped you overcome stigma? Please tell me about your experiences that led you to improve your experience of stigma.

  Probe:

  - *When did it happen? What specific experience/incident/event was involved? How did it make you feel? (Probe further: Tell me more. Can you give an example? Another example?)*

- What happened or did you do that helped you overcome stigma/shame/sadness/misery of having the health condition? Did you have any strategies, made any changes, or received any help which helped you overcome stigma? Please tell us about what strategies, resources, or support helped you overcome stigma?

  Probe:

  - *Tell us what you did – or what happened - or what helped– which may be strategies of overcoming the stigma, being support by people, not feeling ashamed of your condition, being*

able to disclose and talk about your condition, meeting other people with similar condition and working with them, advocating for your rights etc. (Probe further to find out ***the main factors that led them*** *to having the positive deviant strategies of overcoming stigma, being empowered, and being supported by others)*

- *What and/or who has been the most important person or resource in leading to your positive life change and the strategies that helped you? How did it/they help you in developing the strategies? (Probe further: Who or what played an important role? So, tell me more about it. How did it help? Can you give an example? Can you give another example?)*

- Do you feel that the strategies/changes that you used to help overcome stigma also helped in changing other aspects of your daily life? What aspects of your life do you think were affected and how? Please think of how it affects your life, quality of life (physical, mental, social, economic) and interaction with others.

  <u>*Probe:*</u>

  - *What changes did you feel after you overcame stigma?*
  - *How do these changes/strategies affect your life, the quality of life (physical, mental, social, economic)?*
  - *How does it influence your daily interaction with others? (probe: the changes)*
  - *Probe further: Tell me more. Can you give an example? Another example?*

- As of now – after not being ashamed or feeling stigma because of your health condition, do you feel being more included in the society? Please think of how your interaction in the society may have changed. How do you interact in the society now. Tell us of your experiences.

Probe:

- *How well have you had the opportunity to participate in the society- religious, communal, employment, facilities, welfare, education, healthcare, etc. after being open and not feeling ashamed anymore?*
- *Can you tell how you felt to be included in the society? How did you feel included? What happened that made you feel you were included and welcomed in the society, specifically after applying the positive deviant strategy? Can you give examples of your experience of inclusion?*
- *What do you think was important in helping or facilitating your experience of inclusion in the society? What and/or who has been the most important person or resource in leading to your experience of inclusion in the society? How did it/they help you in your experience? (Probe further: What resources or which persons played an important role? So tell me more about this resource or person. How did they help you? Why do you think did this person support you/ included you? Can you give an example? Can you give another example?)*

- How has the feeling of not being ashamed/sad/stigmatized because of your health condition shaped your life? What decisions were shaped because of being able to dispel stigma in your life – which you think would have not happened if you still were stigmatized/ashamed of your health condition? What works or activities are you engaged in these days (with family, friends, society, health services, peer-groups, jobs/career)? What have you planned for the future, and what does the future hold for you?

  Probe:

  - *Think about a scenario where you would have not had these changes and would be still feeling ashamed and stigmatized – compare how your life has changed.*
  - *How did these changes helped you in decision making? What decisions did you make that shaped your life?*

- *Tell me more of the activities you started doing after dispelling stigma and coming out proud. What activities are you engaged in currently.*
- *What are you planning to do in the future? How are you planning to go ahead in regards to your health condition – healthcare, medication (if currently in medication), etc. How are you planning to go ahead in regards to your family and social connection? How are you planning to go ahead in regards to your job or career?*
- *Probe further: Tell me more. Can you give an example? Another example?*
