# Supplementary material for: How do Positive Deviants Overcome Health-Related Stigma? An Exploration of Development of Positive Deviance Among People With Stigmatized Health Conditions in Indonesia
Source: Qual Health Res. 2021 Dec 14;32(4):622–34. doi: 10.1177/10497323211058164 (PMC8853968; doi:10.1177/10497323211058164)
Supplement: sj-pdf-2-qhr-10.1177_10497323211058164 – Supplemental Material for How do Positive Deviants Overcome Health-Related Stigma? An Exploration of Development of Positive Deviance Among People With Stigmatized Health Conditions in Indonesia [file sj-pdf-2-qhr-10.1177_10497323211058164.pdf]

SUPPLEMENTARY FILE 2

Coding process and resultant outcomes : Constructivist grounded theory approach

**Coding Stage**	**Description of activity**	**Outcome**	
**Stage 1:**<br><br>**Initial Coding**	The transcripts were read line-by-line and incident-by-incident coding was conducted within each interview to obtain the initial codes.	A total of **120 codes** were obtained through initial coding.	**MEMO WRITING***
**Stage 2:**<br><br>**Focused Coding**	Codes were compared across all interviews and clustered into categories. As the coding process proceeded, it became clear that some of the categories corresponded to most positive deviants, which became the main theoretical categories. There were some categories that were less mentioned and formed sub-categories. The categories were then grouped into interconnected preliminary themes.	Preliminary themes and categories obtained:<br><br>**Theme 1: Development of Positive deviance**<br><br>(Categories: Self-acceptance, self-confidence, positive outlook, motivation, experiential knowledge, information, situational assessment and awareness, disclosure considerations, support from others, resource mobilization skills, self-care, indifference to stigmatizing views/behaviors, disclosure of status, situational adjustments, leading by example, supporting peers, activism, advocacy)<br><br>**Theme 2: Impact of Positive deviance**<br><br>(Categories: helping others, providing knowledge, community outreach, foster awareness, self-strengthening/reiteration and reinforcement)	
**Stage 3:**<br><br>**Theoretical Coding**	Interrelated categories were further integrated into themes. The connection and	Three themes and associated categories obtained:<br><br>**Theme 1: Triggers**	

	pathway between the themes were discussed. Three main themes and associated categories emerged from theoretical coding. The process of theoretical coding led to a preliminary theoretical framework obtained through assimilation and thematic conceptualization of interrelated categories.	(Categories: Knowledge, motivation, both knowledge and motivation)<br><br>**Theme 2: Empowerment**<br><br>(Categories: Self-acceptance, self-confidence, positive outlook, motivation, experiential knowledge, information, situational assessment and awareness, disclosure considerations, support from others, resource mobilization skills, self-care, indifference to stigmatizing views/behaviors, disclosure of status, situational adjustments, leading by example, supporting peers, activism, advocacy, self-strengthening)<br><br>**Theme 3: Impact**<br><br>(Categories: helping others, providing knowledge, community outreach, foster awareness, reinforcement)	
**Stage 4:**<br><br>**Literature review and application of extant theoretical models**	The resultant theoretical concepts were compared with existing relevant literature in the later stage of data analysis. The theoretical categories pertaining to the theme “empowerment”, based on the similarity, was fitted into existing theoretical model of psychological empowerment by Zimmerman (1995).	Theme 2: Empowerment - fitted into theoretical model of psychological empowerment.<br><br>**New fitted theme: Psychological Empowerment**<br><br>• Intrapersonal component<br>• Interactional component<br>• Behavioral component	
**Stage 5:**<br><br>**Final theoretical**	The main themes were integrated with the extant theoretical model. The themes and categories were re-	Final themes and categories:<br><br>**Trigger**<br><br>- Motivation	

**framework**	evaluated to ensure their connection and pathway. Final theoretical framework was obtained.	- Information<br>**Initiation**<br>- Intrapersonal component (Self-acceptance, self-confidence, positive outlook, motivation)<br>- Interactional component (experiential knowledge, information, situational assessment and awareness, disclosure considerations, support from others, resource mobilization skills)<br>- Behavioral component (self-care, indifference to stigmatizing views/behaviors, disclosure of status, situational adjustments, leading by example, supporting peers, activism, advocacy)<br>**Self-strengthening**<br>**Impact**<br>- Empowering others affected through knowledge diffusion<br>- Foster social awareness and acceptance.<br>**Reinforcement**	

** Memo writing was carried out in every stage of the analysis.*
