# Supplementary material for: How do Positive Deviants Overcome Health-Related Stigma? An Exploration of Development of Positive Deviance Among People With Stigmatized Health Conditions in Indonesia
Source: Qual Health Res. 2021 Dec 14;32(4):622–34. doi: 10.1177/10497323211058164 (PMC8853968; doi:10.1177/10497323211058164)
Supplement: sj-pdf-3-qhr-10.1177_10497323211058164 – Supplemental Material for How do Positive Deviants Overcome Health-Related Stigma? An Exploration of Development of Positive Deviance Among People With Stigmatized Health Conditions in Indonesia [file sj-pdf-3-qhr-10.1177_10497323211058164.pdf]

# SUPPLEMENTARY FILE 3

## Summary of the categories of the three components of psychological empowerment, their description and illustrative quotes

1. **Intrapersonal component**

| Category | Description | Illustration by quote |
|---|---|---|
| Self-acceptance | Positive deviants talked about how accepting themselves and their health condition was the first step to feeling better about themselves and improving self-esteem. | *I accepted my life with diabetes and I am finally in peace with myself. I am now more grateful because I don't feel low anymore. I instead feel good about myself. (Female, Diabetes)* |
| Self-confidence | Having self-confidence was very important for positive deviants to be self-aware of their ability to perceive control of their life. | *Confidence is the key to happiness. I was not embarrassed to talk about my health condition. I could take care of myself and support my family, and at the same time help others. In fact, I became a problem solver for my friends. (Male, Leprosy)* |
| Positive outlook | Positive deviants reported of having positive insights and outlook to life which ignited a sense of hopefulness and normalcy in their lives. | *[…] we need to think positively, and handle ourselves; we don't need to always depend on others if it's our own health problem. Every disease is the same, it's not just schizophrenia, there is also diabetes, leprosy, HIV, disabilities, heart disease. We don't have to undervalue ourselves. We can be active again just like any healthy person. (Male, Schizophrenia)* |
| Motivation | Positive deviants derived motivation from three different sources (described below) that provided them the incentive to keep going on and striving for better. | |
| *Motivation:* Empathy towards others | The first source of motivation came from being able to relate to others living with the same health condition as them and having | *I know it is unpleasant to get discriminated. It was really sad, and I have gone through it so I don't want my friends (others with HIV) to get the same treatment. I feel for my friends (others with HIV), and I will do anything to make sure they don't have the* |

| | | |
|---|---|---|
| | empathy towards them. | *same bad experience as me. (Female, HIV)* |
| *Motivation:* Experience of stigma | A second source of motivation came from experience of stigma in their everyday life from family, friends, and healthcare professionals. | *[…] it [discrimination] came from the doctors themselves. There were one or two doctors in the hospital that specifically worked in HIV care, but others didn't. But sometimes even the doctors specializing in HIV discriminated against us. I knew I did not deserve it - I deserved better! (Male, HIV)* |
| *Motivation:* Concern for loved ones | The third source of motivation came from the concern that PDs had for their loved ones – especially close family members (children, spouse, father, mother, etc.) | *[…] it [HIV transmission] already happened to me, so what can I do next? What was on my mind was how to make sure my baby didn't get HIV as well. That was the only thing in my mind. (Female, HIV)* |

## 2. Interactional component

| **Category** | **Description** | **Illustration by quote** |
|---|---|---|
| Experiential knowledge | Positive deviants reported of how their personal experiences of living with the health condition provided a better understanding of not only their disease but also the context of stigma surrounding them. | *I've already experienced and learnt a lot about schizophrenia and how to live with it peacefully. I have lived with it since 2013 and experienced it every day since then. (Male, Schizophrenia)* |
| Information | Other than personal experiences, positive deviants talked about how gaining relevant knowledge and information, either from other experts or through information resources, helped them understand their disease and think of ways to manage them. | *[…] one has to understand HIV first. We have training and information available, and I was concerned and wanted to learn. I wanted to learn, to know more. […] it is what helped strengthen me to be able to reach this point. (Transgender Female, HIV)* |
| | | *Before I felt like I faced this illness all by myself, then after I joined the community, I learnt a lot of people also suffered the same thing. It turns out 1% of Indonesian right now have schizophrenia […] there are around 2.5 million of Indonesia's population who have schizophrenia […] Sometimes we feel like* |

| | | |
|---|---|---|
| | | *giving up, but there are actually a lot of people who are facing the same situation (Male, Schizophrenia)* |
| Situational assessment and awareness | Positive deviants talked about the importance of having a critical awareness of the environment and the social norms in one's daily life and understanding measures to fit-in as a way to avert stigmatization. | *[…] as long as we don't look like a sick person, other people won't be worried. So how do we take care of our health? Well, we feel tired sometimes, or get sick, but we need to be able to control it. (Male, HIV)* |
| Disclosure considerations | Positive deviants highlighted the importance of understanding the people, their beliefs and specific contexts before disclosing one's health status to others. | *[…] consider first before opening up about your status. Consider who you are opening up to. If you open up about your status to people who lack knowledge about HIV – you might get discriminated against and the stigma will spread. We can open up - but see the audience first and know what are their interests. (Transgender Female, HIV)* |
| Support from others | Positive deviants talked about how perception of support from others, especially family and friends, provide positive reinforcement in their lives. | *My family has always been there for me.So when other people discriminated against me, my family supported and strengthened me. (Male, Leprosy)* |
| Resource mobilization skills | Positive deviants discussed how critical awareness of available resources or ways to accessing different resources is paramount to effective management of their life | *Facebook does help because it connects many people. There are also doctors and experts on Facebook who are leaders in the field. So, I can message these doctors for advice through Facebook or watch their online videos. (Male, Schizophrenia)* |

**3. Behavioral component**

| **Category** | **Description** | **Illustration by quote** |
|---|---|---|
| Self-care | Positive deviants highlighted the importance of caring for themselves for not only effective management and treatment of their disease but also for overall well-being. Self-care | *I've been taking care of myself, helping others at home. I feel as if I've been healed since I've been taking medicines regularly. I can be like this as long as I take my medicines. (Male,* |

| | | |
|---|---|---|
| | entailed both physical/medical care (taking medications regularly, following through with health check-ups, etc.) and spiritual care (meditation, prayers, etc.). | *Schizophrenia)* |
| Indifference to stigmatizing views/behaviors | Positive deviants talked about how they ignored and did not care about what others thought of them or talked about them. By this point, they perceived enough control of their life and environment that the public views did not affect them. | *When I experience stigma/discrimination, I just ignore it because it's my life and I choose how to respond to it. If you want to accept me, then I'll be grateful, but if you don't it's okay too. (Male, HIV)* |
| Disclosure of status | Disclosure of their health status was a very important action for positive deviants, which they said not only helped them feel liberated from shame and stigma associated with their disease, but also helped garner support and understanding from others. | *I told (disclosed my status to) people who are closest to me, because I feel that we (those with HIV)are people pierced by many thorns. So how do we break free from those thorns? […] Every time I tell one person it feels like one thorn has been plucked out, and I feel more relieved. (Male, HIV)* |
| Situational adjustments | Positive deviants talked about how adjusting according to different contexts, cultures, and situations help them avert stigma and judgment in their daily lives. They highlighted the importance of critical awareness of the environment and norms in helping decide the required adjustments needed. | *It's important for us to learn to adjust. For example: if I want to socialize with housewives, I wear an appropriate dress that they feel comfortable with, and if I want to socialize with men and younger people, I wear pants just like them. By making those changes, we respect people and in turn we get respect back. (Transgender Female, HIV)* |
| Leading by example (being a role model) | Having understanding and empathy towards others living with their health conditions, positive deviants discussed how they started helping others by showcasing their own life experiences of living with the condition and mentoring and guiding them. | *I try to set good example and be a role model to others with HIV. I show them that I take medicine regularly because of which I am healthy and productive in the society. They learn from me and they understand that they can also turn their lives around just like how I did. (Male, HIV)* |
| Supporting peers | Other than being a role model/mentor, positive deviants also actively helped others affected through actions like | *I visit newly infected patients in their homes. I motivate them to follow the routine treatment. I talk to them,* |

| | | |
|---|---|---|
| | home visits to track their treatment, emotional support and counseling, connecting/referring them to relevant people within their network, lending money when in need, etc. | *share stories, and listen to their problems. They feel like someone cares for them. They feel motivated to take their medication. (Male, Leprosy)* |
| Activism | Positive deviants talked about their passion to fight for their rights and those of other people affected. They showed aversion to inequality and injustice and actively spoke against those, while demanding equality and justice. | *If there is any discrimination, I will fight it. […] After getting repeatedly discriminated by the hospital staffs I ended up getting really angry. I started a protest with other friends and also contacted the media. We need to get accepted; we know the right procedures to do these things. (Female, HIV)* |
| Advocacy | Some positive deviants reported of going a step further ahead from activism towards advocacy where they systematically worked with different stakeholders on affecting policy changes to improve the condition of people living with the health condition as them. | *I'm doing advocacy on sex workers to be mentioned in minimum (health) services standard. It was not there at first, so we've been advocating for it. We went to meetings and it was agreed that sex workers and MSM (men who have sex with men) would be mentioned in the 10 minimum (health) service standard advice, which will soon be implemented. (Male, HIV)* |
